# Exploring inter-rater reliability and measurement properties of environmental ratings using kappa and colocation quotients

**DOI:** 10.1186/1476-069X-13-86

**Published:** 2014-10-23

**Authors:** Jonas Björk, Ralf Rittner, Ellen Cromley

**Affiliations:** Division of Occupational and Environmental Medicine, Lund University, SE-221 85 Lund, Sweden; Department of Community Medicine and Health Care, University of Connecticut School of Medicine, 263 Farmington Avenue, Farmington, CT 06030-6325 USA

**Keywords:** Epidemiologic methods, Spatial analysis, Reproducibility of results, Statistics as topic, Perceived greenness

## Abstract

**Background:**

Available evidence suggest that perceptions or ratings of the neighborhood, e.g. as being green, walkable or noisy, are important for effects on health and wellbeing, also after controlling for objective measures of identical or similar features. When evaluating effects of the perceived environment, it is important that measurement properties and the reliability of the environmental ratings are evaluated before decisions about how these ratings should be handled in the statistical analyses are made. In this paper we broaden the usage of two association measures, the well-known kappa statistic and the novel colocation quotient (CLQ), to studies of inter-rater reliability and of associations between different categorical ratings in spatial contexts.

**Methods:**

We conducted reliability analysis of a survey instrument for assessing perceived greenness at geographical point locations, here the close outdoor environment within 5–10 minutes walking distance from home. Data were obtained from a public health survey conducted in 2008 in Scania, southern Sweden (n =27 967 participants).

**Results:**

The results demonstrate the usefulness of kappa and CLQ as tools for assessing reliability and measurement properties of environmental rating scales when used at geographical point locations. We further show that the two measures are interchangeable, i.e. kappa can be accurately approximated from CLQ and vice versa, but can be used for somewhat different purposes in reliability analyses. Inter-rater reliability between the nearest neighbors was demonstrated for all five items of the evaluated instrument for assessing perceived greenness, albeit with clear differences across the items.

**Conclusion:**

Reliability analysis employing kappa and CLQ can be used as a basis for informed decisions about, for instance, how dichotomizations of the ratings should be defined and how missing or indefinite ratings should be handled. Such reliability analyses can thus serve as guidance for subsequent epidemiological studies of associations between environmental ratings, health and wellbeing.

**Electronic supplementary material:**

The online version of this article (doi:10.1186/1476-069X-13-86) contains supplementary material, which is available to authorized users.

## Background

Perceptions and ratings of environmental attributes have been used extensively in studies of green environments and health [1–4], but also in studies of neighborhood resources for physical activity [5–7], traffic noise [8] and air quality [9, 10]. There is evidence suggesting that perceptions of the neighborhood, e.g. as being green, walkable or noisy, are important for effects on health and wellbeing, also after controlling for objective measures of identical or similar features [1, 6, 8, 11, 12].

Comparisons of self-rated and objectively measured neighborhood attributes show associations (correlations) but generally low agreement [11, 13–16]. The low agreement has been explained as a mismatch between perceptions and objective facts [11], but may also suggest that perceived and objective measures are capturing different aspects of the local environment [13]. However, the rating instruments may lack inter- or intra-rater reliability, thereby contributing to the low agreement with objectively measured attributes. Additionally, decisions about how to collapse or dichotomize categorical ratings, and how to handle indefinite or missing ratings, are often taken arbitrarily without an appropriate analysis of how the ratings are distributed and clustered spatially. Ignoring the spatial distribution of the ratings may add to the discrepancy between exposure measures based on perceptions and objective attributes.

Reliability of individual items in categorical assessment instruments is often evaluated by comparing observed with chance-expected agreement using the kappa statistic, either for a fixed set of raters [17] or for different sets of raters [18] for each assessed object or environment. Kappa is normally used as an overall measure of agreement for an entire rating instrument, but can also be calculated for the agreement between specific categories [18, 19]. For ordinal scales, weights can be applied when calculating kappa to quantify the extent of the disagreement [19, 20].

The colocation quotient (CLQ) was introduced recently as a measure of spatial association among observations of a single categorical variable at point locations [21]. The CLQ builds on the concept of the location quotient, a widely used spatial measure of concentration in a location relative to a norm [22]. While the original purpose of the CLQ was to assess spatial associations between different categories (different classes of objects), it can also be used in analyses of spatial segregation, a phenomenon that occurs when members or ratings of the same category cluster spatially [23]. In the context of environmental ratings at point locations, clustering of identical or compatible ratings will be evidence of inter-rater agreement, i.e. that neighboring subjects agree on the quality of the environment they are rating. When such rating data are obtained from survey questions and the residential locations of the survey participants are known, the inter-rater agreement and reliability can be assessed by comparing the ratings of each participant with the first-order nearest neighbors. Calculated from a nearest neighbor contingency table [23], the CLQ will reflect the inter-rater reliability as a measure of how many times more likely than chance it is that the same categorical rating occurs among the neighbors. Kappa can also be calculated from the same table, providing an opportunity to compare kappa and CLQ as measures of inter-rater reliability of environmental ratings.

The focus in reliability studies in spatial contexts is often on test-retest reliability and internal consistency [5, 24], whereas the reliability across individuals rating the same environment has received much less attention. In the present study we develop the usage of both kappa and CLQ further for assessment of inter-rater reliability. We also explore how these measures can be used to investigate basic measurement properties of the rating scales, appropriate for identification of thresholds for dichotomizations and for decisions about how to handle indefinite or missing ratings. Reliability analysis of perceived greenness in the neighborhood environment will serve as an empirical example throughout the text.

## Methods

### Survey data on environmental ratings

In an extensive cross-sectional public health survey conducted in 2008 in Scania, southern Sweden, n = 27 967 participants with a valid residential address rated the Scania Green Score, which covers five different aspects of the close natural outdoor environment within 5–10 minutes walking distance from home: serenity, wildness, species richness, spaciousness and cultural history (see Additional file 1 for phrasing of the questions) [1, 14]. Each of these five items was rated on a 4-graded ordinal scale: *1 = Disagree completely, 2 = Disagree, 3 = Agree, 4 = Agree completely*. There was also a fifth option, *5 = Do not know/cannot say*, and a sixth value, *6 = Missing answer*. We geocoded the residential address of each (index) participant and identified the first-order nearest neighbors within a specified maximum radius 500 meters in the main analyses. Each index participant and nearest neighbors within 500 meters were assumed to have rated overlapping neighborhood environments in the reliability analyses. Participants without a neighbor within 500 meters (n = 1 757) were thus excluded. This left n = 26 210 sets of index participants matched with their nearest neighbors for analysis. The median distance to the nearest neighbor in this data set was 35 meters (2.5 – 97.5 percentiles 0–330 meters). The distance to the nearest neighbor was generally longer in the semi-urban and rural areas of the study region. As a sensitivity analysis, we also evaluated three other maximum radiuses when identifying the first-order nearest neighbors: 50 meters (n = 15 570), 100 meters (n = 21 230) and 1000 meters (n = 27 364 matched sets included in the analysis). In most cases, participants had a single nearest neighbor, but sometimes more than one participant was located at the same nearest neighbor distance. This typically occurred if participants lived at the same location (e.g., lived in the same multistory building), but it could also occur if residences of different neighboring participants happened to be the same distance away from the residence of the index participant.

### Framework for reliability analysis

We consider a general setting with environmental ratings on a scale with *c* categories, possibly including indefinite and missing answers as separate categories. We assess the agreement between environmental ratings of each matched set *i* (i = 1, 2, 3, …, *n*) of one index participant and its *m*_*i*_ first-order nearest neighbors. The overall proportion of ratings across all index participants in category *j* (*j* = 1, 2, 3, …, *c*) is *p*_*j*_ such that . Let *x*_*i*_(*j*, *k*) denote the number of ratings in set *i* for index rating *j* and nearest neighbor rating *k*. The observed agreement proportion between a specific index rating *j* = *a* and the nearest neighbor rating *k* = *b* can be calculated across all sets of ratings as
1

where 1/*m*_*i*_ is used as a statistical weight to allow for inclusion of all first-order nearest neighbors if more than one neighbor is equally distant from the index participant [21]. Thus, after weighting, each match set will effectively contribute as though they contained two raters (the index rater and one nearest neighbor).

The kappa statistic [18] can be calculated specifically for the reliability of the same categorical rating *a* as
2

where the overall observed agreement proportion between the index participants and the nearest neighbors is


We assume that the nearest neighbors have the same overall distribution of ratings across categories as the index participants to calculate the chance-expected index-neighbor agreement under the null hypothesis of no agreement as


**Figure 1:**
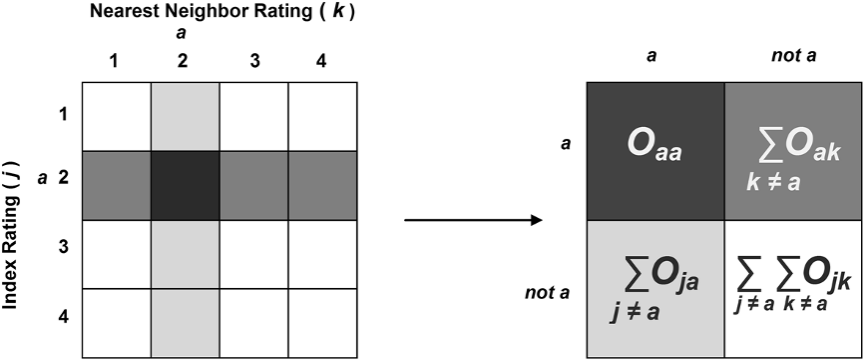
**Converting the matrix of individual by nearest neighbor ratings to measure association for a specific rating.** For a specific rating *a* on a 4-category scale, all individual ratings are collapsed into a 2 × 2 table of *a*/not *a* ratings. Kappa is calculated from the compressed table.

The calculation of kappa for the specific rating *a* in Equation 2 is equivalent to first dichotomizing the rating scale as *a* vs. ¬*a* (“not *a*”) and then calculating the overall kappa of the resulting 2×2-table (Figure 1).

### Calculation and interpretation of kappa

**Figure 2:**
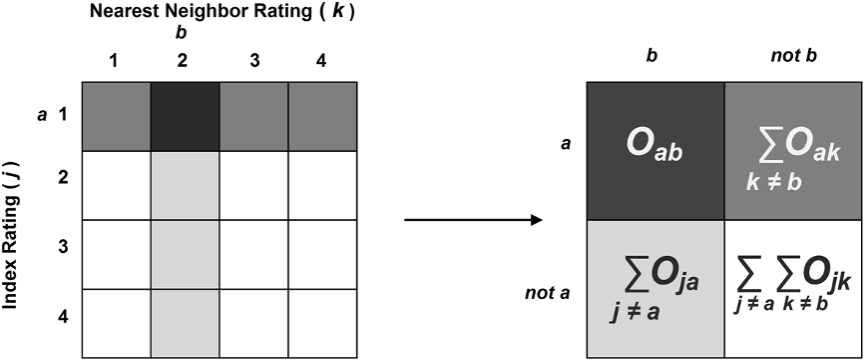
**Converting the matrix of individual by nearest neighbor ratings to measure association for different ratings.** For two different ratings *a* and *b* on a 4-category scale, all individual ratings are collapsed into a 2 × 2 table of *a*/not *a* versus *b*/not *b* ratings. Kappa is calculated from the compressed table.

Kappa can also be extended to measure the association between different index-neighbor ratings *a* and *b*3

where the observed agreement proportion is


and the chance-expected agreement is *P*_*E*_(*a*, *b*) = *p*_*a*_*p*_*b*_ + (1 − *p*_*a*_)(1 − *p*_*b*_) (Figure 2).

In words, kappa reflects how much the observed agreement exceeds chance-expected agreement, *P*_*O*_(*a*, *b*) − *P*_*E*_(*a*, *b*), expressed as a fraction or as a percentage of the maximum excess agreement, 1 − *P*_*E*_(*a*, *b*), that is possible to obtain. A kappa value of zero would imply no more than chance-expected agreement. When kappa is positive, observed agreement exceeds the chance-expected agreement (positive agreement). When kappa is negative, observed agreement is lower than chance-expected (negative agreement).

### Calculation and interpretation of CLQ

We modify the original formulation of the CLQ [21] for the association between rating *a* and *b* to allow for sampling with replacement, i.e. the index rating is included in the calculation of the chance-expected agreement. As such, the CLQ can be calculated as:
4

where *o*_*ab*_ is the observed agreement proportion (colocation) between index rating *a* and nearest neighbor rating *b* as defined in Equation 1 and *p*_*a*_ · *p*_*b*_ is the chance-expected colocation. CLQ values range from 0 to a theoretical maximum that is dependent on the relative counts *p*_*a*_ and *p*_*b*_ as well as on certain geometrical constraints [21]. To get the theoretical maximum of CLQ we condition on the total proportion of ratings *p*_*a*_ + *p*_*b*_ in category *a* and *b*. By assuming equal occurrence of *a* and b, , the maximum of CLQ can be obtained as
5

A CLQ value of one would imply no more than chance-expected agreement. For evidence of spatial association (positive agreement) between *a* and *b*, we would want to see CLQ values above 1, i.e. a higher number of nearest neighbors with *b* ratings than expected given its relative count in the population. When CLQ is below one, the observed colocation between *a* and *b* is less than expected (negative agreement). The lowest possible value of CLQ is zero, which only occurs if no nearest neighbor of the *a* ratings has rated the environment as *b*. Note that both kappa and CLQ are asymmetric measures, i.e. *CLQ*(*a*, *b*) is not necessarily equal to *CLQ*(*b*, *a*).

### Comparisons of applicability of kappa and CLQ

To facilitate comparison between kappa and CLQ, we can use that the observed agreement proportion *P*_*O*_(*a*, *b*) between category *a* and *b* can be approximated by the following formula


(identity holds when the nearest neighbors and index participants have identical overall relative counts across category *a* and *b*, i.e. when ∑*o*_*jb*_ = *p*_*b*_ and ∑*o*_*ak*_ = *p*_*a*_). Using the expressions for CLQ (Equation 4) and *CLQ*_*Max*_ (Equation 5), kappa can be approximated as
6

In words, we can always normalize CLQ to an approximate kappa by subtracting the CLQ with one and dividing with its theoretical maximum minus one. With positive agreement for a specific categorical rating *a* the CLQ will reflect a multiple of agreement more than expected, whereas kappa in percent will reflect how large the excess agreement is in relation to the maximum excess in agreement that can be obtained.

We present the reliability results below by reporting the CLQ together with kappa in percent as ‘CLQ (kappa %)’. For associations between different categorical ratings *a* and *b*, CLQ and kappa will yield information about which ratings (including indefinite or missing ratings) cluster (CLQ > 1; Kappa > 0) or separate (CLQ < 1; Kappa < 0) in space. CLQ and kappa can thus be used not only to assess inter-rater reliability but also for assessing other basic measurement properties of rating scales. The results can for example serve as a basis for decisions about collapsing categories on the rating scale and for decisions about how to handle indefinite or missing ratings. We used the following three criteria, based on CLQ or kappa or both measures, to identify tentatively pairs of categorical ratings that could be combined without loss of reliability: i) a spatial clustering (CLQ > 1; Kappa > 0) between the two ratings; ii) a CLQ that was of the same magnitude as the lowest of the two corresponding same-category CLQs; and iii) an increase in the same-category kappa of the collapsed vs. original ratings.

### Statistical analysis

All statistical analyses were performed in R version 3.0.1 [25]. The distance calculations used in order to identify nearest neighbors were done with the *fields* package for spatial data version 6.9.1, function *rdist* [26]. We used the approximate standard error (SE) expression derived by Fleiss and Cohen for kappa between two raters [27] to assess tentatively the empirical evidence for more than chance-expected agreement (positive or negative) between category *a* and *b*:


## Results

Reliability analysis was conducted for all five items of the Scania Green Score using the first-order nearest neighbors within the radius 500 meters in the main analyses. Results for three of the items, species richness, wildness and cultural history, showed similarities, whereas the results of the two remaining items, serenity and spaciousness, were different but similar to each other. We therefore focus on one item in each group, species richness and serenity, to illustrate how CLQ and kappa can be used when exploring inter-rater reliability and measurement properties (Tables 1, 2 and 3). Results for the three other items are presented in Additional file 2: Tables S1-S3. It should also be noted that both CLQ and kappa exhibited symmetry, i.e. similar associations between *a* and *b* as between *b* and *a*, across all five items, although that need not always be the case with other spatial distributions of ratings. Due to the symmetry we will therefore limit the presentation in the contingency tables to results above the diagonals from top-left to bottom-right.

**Table 1:** **Index – nearest neighbor rater agreement on species richness in the close outdoor environment**

	Rel	Nearest neighbor rater											
	freq^a^	1. Disagree completely		2. Disagree		3. Agree		4. Agree completely		5. Cannot say		6. Not answered	
	(%)	Obs/Exp^b^	CLQ (K %)	Obs/Exp^b^	CLQ (K %)	Obs/Exp^b^	CLQ (K %)	Obs/Exp^b^	CLQ (K %)	Obs/Exp^b^	CLQ (K %)	Obs/Exp^b^	CLQ (K %)
Index rater													
1 Disagree completely	13	2.7/1.6	1.7 (9.5)^***^	4.9/3.9	1.3 (4.9)^***^	3.1/4.3	0.72 (−6.2)^***^	0.50/1.3	0.33 (−8.8)^***^	0.95/0.90	1.1 (1.0)	0.62/0.54	1.2 (1.3)
2 Disagree	31			12/9.3	1.3 (11)^***^	8.6/10	0.84 (−7.3)^***^	1.7/3.6	0.47 (−11)^***^	2.3/2.1	1.1 (1.2)	1.3/1.3	0.99 (0.0)
3 Agree	34					13/11	1.2 (8.8)^***^	5.1/4.0	1.3 (6.6)^***^	2.1/2.4	0.88 (−1.3)	1.3/1.4	0.90 (−0.7)
4 Agree completely	12							3.4/1.4	2.5 (20)^***^	0.52/0.83	0.63 (−3.1)^*^	0.40/0.50	0.79 (−1.1)
5 Cannot say	7.0									0.70/0.49	1.4 (3.8)^*^	0.36/0.29	1.2 (1.6)
6 Not answered	4.2											0.26/0.18	1.5 (2.5)

*0.01≤ p <0.05; ***p <0.001.

^a^Relative frequency of index ratings in each category.

^b^Observed/Expected relative frequency (%) used in the calculation of the colocation quotient (CLQ).

**Table 2:** **Index – nearest neighbor rater agreement on species richness collapsed into a 3 × 3-table**

	Rel	Nearest neighbor rater					
	freq^a^	1-2 Disagree completely/Disagree		3-4 Agree/Agree completely		5-6 Cannot say/Not answered	
	(%)	Obs/Exp^b^	CLQ (K %)	Obs/Exp^b^	CLQ (K %)	Obs/Exp^b^	CLQ (K %)
Index rater							
1-2 Disagree completely/Disagree	43	24/19	1.3 (21)^***^	14/19	0.71 (−23)^***^	5.1/4.8	1.1 (1.5)^*^
3-4 Agree/Agree completely	46			27/21	1.3 (26)^***^	4.3/5.1	0.84 (−3.3)^***^
5-6 Cannot say/Not answered	11					1.7/1.3	1.3 (4.9)^***^

*0.01≤ p <0.05; ***p <0.001.

^a^Relative frequency of index ratings in each category.

^b^Observed/Expected relative frequency (%) used in the calculation of the colocation quotient (CLQ).

**Table 3:** **Index – nearest neighbor rater agreement on serenity in the close outdoor environment**

	Rel	Nearest neighbor rater											
	freq^a^	1. Disagree completely		2. Disagree		3. Agree		4. Agree completely		5. Cannot say		6. Not answered	
	(%)	Obs/Exp^b^	CLQ (K %)	Obs/Exp^b^	CLQ (K %)	Obs/Exp^b^	CLQ (K %)	Obs/Exp^b^	CLQ (K %)	Obs/Exp^b^	CLQ (K %)	Obs/Exp^b^	CLQ (K %)
Index rater													
1 Disagree completely	5.7	0.60/0.32	1.9 (5.9)^***^	1.6/10	1.6 (4.6)^***^	2.2/2.4	0.91 (−1.1)	0.77/1.6	0.49 (−4.8)^***^	0.29/0.20	1.5 (1.9)	0.25/0.20	1.3 (1.7)
2 Disagree	18			4.8/3.1	1.5 (11)^***^	7.3/7.3	0.99 (−0.2)	2.5/4.9	0.51 (−13)^***^	0.80/0.62	1.3 (1.7)	0.67/0.62	1.1 (0.7)
3 Agree	42					19/17	1.1 (4.6)^***^	11/12	0.92 (−3.7)^***^	1.5/1.5	1.0 (−0.0)	1.4/1.5	0.95 (−0.2)
4 Agree completely	28							12/7.8	1.6 (23)^***^	0.56/0.98	0.57 (−2.9)^**^	0.79/0.98	0.80 (−1.2)
5 Cannot say	3.5									0.21/0.12	1.7 ( 2.2)	0.17/0.12	1.4 (2.2)
6 Not answered	3.5											0.20/0.12	1.6 (3.0)

**p <0.01; ***p <0.001.

^a^Relative frequency of index ratings in each category.

^b^Observed/Expected relative frequency (%) used in the calculation of the colocation quotient (CLQ).

Among the index raters, 34% rated species richness in their neighborhood as *3 = Agree* and 12% rated species richness as *4 = Agree completely* (Table 1). There was clear evidence (p < 0.001) of index-nearest neighbor reliability, i.e. observed agreement differing significantly from the chance-expected, for each of the informative ratings 1–4 as shown in the top-left to bottom-right diagonal of Table 1 with CLQs ranging between 1.2 and 2.5. A CLQ of 2.5 as for rating 4 implies that the same rating among the nearest neighbors as among the index raters was 2.5 times as likely as could be expected by chance (observed agreement 3.4% vs. expected agreement 1.4%; Table 1). These CLQ values correspond to 8.8%-20% of maximum agreement when expressed as kappa (Table 1). There was clear evidence (p < 0.001) of positive agreement between the ratings *1 = Disagree completely* and *2 = Disagree* [CLQ (kappa %) = 1.3 (4.9%)] and between 3 and 4 [CLQ (kappa %) = 1.3 (6.6%)], and these across-categories CLQs were of the same magnitude as the lowest of the corresponding same-category CLQs. On the other hand, the agreements between 1–3, 1–4, 2–3 and 2–4 were all negative with CLQs below one (CLQ range 0.33 – 0.84; p < 0.001). Taken together, these results suggest a spatial association between 1–2 (*Disagree completely-Disagree*) and between 3–4 (*Agree-Agree completely*) and that an appropriate threshold for dichotomization among the informative ratings 1–4 would be between category 2 and 3.

The proportion of indefinite (5) or missing ratings (6) on species richness was 11.2% (7.0% +4.2%) among the index raters (Table 1). Indefinite and missing ratings did not seem to be distributed randomly (CLQ with the same rating was 1.4 and 1.5, respectively; Table 1), although the statistical support for these associations was not consistently strong (p = 0.02 for rating 5 and p = 0.23 for rating 6). The CLQ between 5 and 6 was somewhat lower (1.2) than the two corresponding same-category CLQs and statistically uncertain (p > 0.30). Spatial separation between ratings 5–6 and 3–4 with CLQs consistently below one was noted, but generally with substantial statistical imprecision inherent. Thus, there was some, but statistically weak, evidence suggesting that collapsing ratings 5 and 6 could be appropriate.

The consequences of collapsing ratings 1–2, 3–4 and 5–6 are presented in Table 2. The inter-rater reliability of the collapsed ratings 1–2 (Disagree completely/Disagree) and 3–4 (Agree/Agree completely) expressed as kappa increased from 9.5%/11% to 22% for ratings 1–2 and from 8.8%/20% to 26% for ratings 3–4. Furthermore, the collapsed ratings 1–2 and 3–4 were clearly separated [CLQ (kappa %) = 0.71 (−23%); p < 0.001], providing additional support for combining the ratings in this way. Collapsing ratings 5–6 (Cannot say/Not answered) increased kappa from 3.8%/2.5% to 4.9% (p < 0.001 for kappa within ratings 5–6). Ratings 5–6 were more clearly separated from ratings 3–4 when combined [CLQ (kappa %) = 0.84 (−3.3%); p < 0.001], but showed only weak spatial association with the collapsed ratings 1–2 [CLQ (kappa %) = 1.1 (1.5%), p = 0.03]. Thus, a further grouping of ratings 5–6 with ratings 3–4 would be clearly inappropriate. On the other hand, there is no strong support for combining ratings 5–6 with ratings 1–2.

The serenity of the neighborhood environment was rated as *4 = Agree completely* by 28% and as *3 = Agree* by 43% of all index raters (Table 3). Index-nearest neighbor agreement was suggested for each of the informative ratings 1–4 as shown in the diagonal of Table 3 with CLQs ranging between 1.1 and 1.9, corresponding to 4.6 - 23% of maximum agreement when expressed as kappa. There was positive agreement between rating 1 and 2 [CLQ (kappa %) = 1.6 (4.6%)]; p < 0.001 with the CLQ being compatible with the lowest of corresponding two same-category CLQs (1.5), whereas a weak spatial separation between rating 3 and 4 was observed [CLQ (kappa %) = 0.92 (−3.7%), p < 0.001; Table 3]. There was clear evidence of spatial separation between rating 1 and 4 [CLQ (kappa %) = 0.49 (−4.8%); p < 0.001] and between 2 and 4 [CLQ (kappa %) = 0.51 (−13%); p < 0.001], while no apparent association was detected between 1 and 3 or between 2 and 3. These results thus suggest a spatial colocation of rating 1 and 2 while rating 3 and 4 seem to be separate entities. As for species richness, the spatial association with indefinite (5) and missing ratings (6) was somewhat lower (1.4) than the two corresponding same-category CLQs (1.7 and 1.6, respectively; Table 3) and statistically uncertain. A negative association between rating 4 and 5 was observed [CLQ (kappa %) = 0.57 (−2.9%), p = 0.002].

The consequences of collapsing ratings 1–2 and 5–6 are presented in Table 4. The inter-rater reliability of the collapsed ratings 1–2 (Disagree completely/Disagree) and 5–6 (Cannot say/Not answered) expressed as kappa increased from 5.9%/11% to 17% for ratings 1–2 and from 2.2%/3.0% to 4.4% for ratings 5–6. By contrast, collapsing ratings 3–4 (Agree/Agree completely) would not increase the reliability: kappa = 19% for ratings 3–4 combined (not in tables) vs. 4.6%/23% for rating 3 and 4 as separate categories (Table 4).

**Table 4:** **Index – nearest neighbor rater agreement on serenity in the close outdoor environment**

	Rel	Nearest neighbor rater							
	freq^a^	1 - 2. Disagree completely/Disagree		3. Agree		4. Agree completely		5 - 6. Cannot say/Not answered	
	(%)	Obs/Exp^b^	CLQ (K %)	Obs/Exp^b^	CLQ (K %)	Obs/Exp^b^	CLQ (K %)	Obs/Exp^b^	CLQ (K %)
Index rater									
1-2 Disagree completely/Disagree	23	8.5/5.4	1.6 (17)^***^	9.5/9.7	0.97 (−1.2)	3.3/6.5	0.51 (−16)^***^	2.0/1.6	1.2 (2.9)^**^
3 Agree	42			19/17	1.1 (4.6)^***^	11/12	0.92 ( −3.7)^***^	2.9/2.9	0.98 (−0.2)
4 Agree completely	28					12/7.8	1.6 (23)^***^	1.4/2.0	0.68 (−3.9)^***^
5-6 Cannot say/Not answered	7.0							0.77/0.49	1.6 (4.4)^**^

**p <0.01; ***p <0.001.

^a^Relative frequency of index ratings in each category.

^b^Observed/Expected relative frequency (%) used in the calculation of the colocation quotient (CLQ).

The presented kappa values were calculated from the definition in Equation 3, but can also be approximated closely by normalizing the corresponding CLQ using Equation 6. As an example, the reported kappa between rating 3 and 4 for species richness was 0.066 = 6.6%, which can also be obtained approximately from CLQ and the relative counts in Table 1 as


In the sensitivity analysis, an increased maximum radius when identifying the first-order nearest neighbors was associated with the inclusion of more rural environments and thereby larger proportions of positive assessments of neighborhood greenness (not in tables). If anything, the reliability increased somewhat with larger radiuses. As an example, the kappa for species richness, rating 4, increased from 18% with radius 50 meter (8.5% of all included index ratings) to 22% with radius 1 000 meter (13.1% of all included index ratings). It thus seems that the neighbor environments in the rural areas were easier to agree on, despite less spatial overlap (i.e. larger distance to the nearest neighbors) for those environments.

## Discussion

Our empirical example demonstrates the usefulness of kappa and CLQ as tools for assessing reliability and measurement properties of environmental rating scales when used at geographical point locations. The two measures are interchangeable, i.e. kappa can be accurately approximated from CLQ and vice versa, but can as seen in our example be used for somewhat different purposes in the reliability analysis. CLQ is a measure of spatial association and was used to identify sets of ratings with compatible colocation across as within categories, whereas kappa is a measure of agreement and was used to assess effects on reliability when clustered sets of ratings were combined. An advantage of kappa is that it is a well-known measure of agreement in other (non-spatial) settings within epidemiology and clinical research. The present study broadens the usage of kappa to studies of reliability and of associations between different categorical ratings in spatial contexts. By contrast, the novel CLQ is an explicit spatial measure that requires information on the spatial arrangement of ratings. CLQ is designated to show the level of spatial association among each pair of ratings and has its strength in the appealing interpretation of how many more times more likely concordant ratings and observed colocations are than chance. When there is interest in exploring the spatial structure of agreement in environmental ratings, CLQ may be the preferred approach.

Inter-rater reliability was in the present study demonstrated for all five items of the Scania Green Score, albeit with clear differences across the items. Previous studies using these items have dichotomized the scale as *Disagree completely* or *Disagree* vs. *Agree* or *Agree completely* [1, 14]. The present results provide support for treating the scale in this way only for three of the items, species richness, wildness and cultural history. By contrast, the analysis of the two other items, serenity and spaciousness, provided no firm support for dichotomizations. Furthermore, indefinite and missing answers have in previous studies been combined with *Disagree completely*/*Disagree* [1, 14], but the present reliability analysis only showed a weak spatial association that would motivate this. It should be noted that we restricted the attention to reliability issues when different groupings of the original ratings were considered. In practice, validity issues should also be addressed, e.g. environmental ratings that reflect different underlying constructs might be inappropriate to collapse even if they exhibit colocation. The consequence of inappropriate collapsing of environmental ratings would be equivalent to non-differential misclassification of exposure. Such misclassification yields bias towards the null if the final classification used in the epidemiological analysis is dichotomous, whereas the resulting bias is less predictable in case of a polychotomous classification [28].

The large survey sample, with 26 210 sets of index participants matched with their nearest neighbors, made it possible to conduct detailed reliability and association analyses with sufficient statistical precision for the informative ratings 1–4, whereas the statistical power to detect spatial associations with the less prevalent indefinite and missing ratings was lower. Another study limitation was that the index-neighbor ratings did not refer to the exactly the same neighborhood environment. Although the distance between the residences within the matched sets of raters was generally short, differences in the geographical point locations probably contributed to dispersion in the ratings. When designing reliability studies of environmental ratings, it is essential that the geographical sampling is such that sufficient numbers of participants are rating the same environments.

Kappa and CLQ differ with respect to their maximum values. Kappa is normalized and its maximum is therefore always 100% when expressed as a percentage. By contrast, CLQ is not normalized; the maximum value will be higher the lower the relative frequency of the least common of the two ratings is. Maximum CLQ will also be higher the more similar the relative frequencies of the two ratings are. Thus, it is not obvious to judge how impressive a CLQ of, say, 1.5, is, unless the corresponding kappa value is also presented. In our empirical example, the kappa ranged between 2% and 11% for different CLQs of 1.5. For this reason, we used the change in kappa rather than in CLQ to assess the effect on reliability when rating categories were combined. For a given agreement proportion, both kappa and CLQ will be dependent on the number of categories and marginal frequencies of the evaluated contingency table. This is not necessarily a weakness of the measures per se, since identical agreement proportions should be judged differently depending on the chance-expected agreement [29]. Thus, when comparing reliability and spatial associations across study settings, the concern should not be differences in marginal frequencies as such, but rather differences in the proportion of local environments that are difficult to decide on [29].

A concern with the kappa measure, which also applies for the CLQ, is that it does not distinguish between systematic and random disagreement [30]. However, this was less of a problem in our empirical example where index and nearest neighbor had similar overall distributions of ratings, suggesting that the disagreement is random rather than systematic. The reported kappa values (at most 10-21% when calculated between the same informative ratings 1–4 of an item) may seem low compared with usual requirements for sufficient inter-rater agreement in other settings. An important explanation for the low agreement, besides differences in the geographical point locations, is likely to be factors on the individual-level related to socioeconomy, e.g. housing situation, country of origin and education, which have been shown to influence the perception of the neighborhood [31]. The influence of such individual factors may bias epidemiological associations between individual ratings of the environment and health outcomes if not adjusted for [14]. One way of improving the generalizability of the individual ratings, as well as limiting the bias, would be to aggregate all ratings within a neighborhood [9, 14, 24]. However, the results of the present study imply that such aggregations should not be conducted without firstly analyzing reliability and measurement properties of the ratings at the individual-level. Further matching for factors known to influence perception of the neighborhood could be considered in reliability analyses but would limit generalizability of the results and, at least in the present study, decrease sample size.

The present study only assessed reliability and colocation globally, i.e. only for the entire survey cohort, and thereby ignoring much of the spatial context. For simplicity reasons, all matched sets obtained equal statistical weight in the analyses, regardless of the number of and distance to nearest neighbors. The CLQ was recently extended to a local counterpart using principles of geographical weighting based on the distance between the objects [32]. A natural extension of the present study would therefore be to extend the analysis spatially, aiming at identifying individual-level or local geographical determinants of the reliability and colocation. We only assessed the statistical uncertainty of the suggested spatial associations tentatively; developing and validating formulas for the standard error is an issue that also deserves further attention.

In conclusion, both the extended definition of the well-known kappa statistic and the novel CLQ were shown to be appropriate tools for investigating inter-rater reliability and spatial associations between environmental assessments on categorical ratings scales. Such reliability analyses should serve as guidance for subsequent epidemiological studies of associations between environmental ratings, health and wellbeing.

## Electronic supplementary material

Additional file 1: **Survey questions in Scania Green Score.** (DOC 51 KB)

Additional file 2: Table S1: Index – nearest neighbor rater agreement on wildness in the close outdoor environment. **Table S2.** Index – nearest neighbor rater agreement on spaciousness in the close outdoor environment. **Table S3.** Index – nearest neighbor rater agreement on cultural history in the close outdoor environment. (DOC 106 KB)

## Abbreviation

CLQ: Colocation quotient.
