# Supplementary material for: Exploring inter-rater reliability and measurement properties of environmental ratings using kappa and colocation quotients
Source: Environ Health. 2014 Oct 23;13:86. doi: 10.1186/1476-069X-13-86 (PMC4223848; doi:10.1186/1476-069X-13-86)
Supplement: Supplementary file 1 — Additional file 1: Survey questions in Scania Green Score. (DOC 51 KB) [file 12940_2014_793_MOESM1_ESM.doc]

**Additional File 1 - Survey questions in Scania Green Score**

Each of the five items below corresponds to a specific aspect of the natural outdoor environment
a) serenity, b) wildness, c) species richness, d) spaciousness and e) cultural history.

*Think of nature within 5-10 minutes walking distance from where you live. This can for example be green spaces, parks or forest areas. Do you agree with the following statements? Choose one option for each line!*

Nature in the area where I live *Disagree Disagree Agree Agree Do not know/*

*completely completely Cannot say*

1. is serene, one can hear nature’s

own sound

1. is wild, it has developed without

human impact

1. has a large diversity of animal

and plant species

1. is a large cohesive area
2. makes you feel the historical

heritage, for example ancient

monuments, old trees, constructions
