# Supplementary material for: Exploring inter-rater reliability and measurement properties of environmental ratings using kappa and colocation quotients
Source: Environ Health. 2014 Oct 23;13:86. doi: 10.1186/1476-069X-13-86 (PMC4223848; doi:10.1186/1476-069X-13-86)
Supplement: Supplementary file 2 — Additional file 2: Table S1: Index – nearest neighbor rater agreement on wildness in the close outdoor environment. Table S2. Index – nearest neighbor rater agreement on spaciousness in the close outdoor environment. Table S3. Index – nearest neighbor rater agreement on cultural history in the close outdoor environment. (DOC 106 KB) [file 12940_2014_793_MOESM2_ESM.doc]

**Additional File 2 – Table S1 – S3**

**Table S1. Index – nearest neighbor rater agreement on wildness in the close** outdoor environment

|  | Rel | Nearest neighbor rater | | | | | | | | | | | | | | | |
| --- | --- | --- | --- | --- | --- | --- | --- | --- | --- | --- | --- | --- | --- | --- | --- | --- | --- |
|  | freq^a^ | 1. Disagree completely | | 2. Disagree | | 3. Agree | | | 4. Agree completely | | | 5. Cannot say | | | 6. Not answered | | |
|  | (%) | Obs/Exp^b^ | CLQ (K %) | Obs/Exp^b^ | CLQ (K %) | | Obs/Exp^b^ | CLQ (K %) | | Obs/Exp^b^ | CLQ (K %) | | Obs/Exp^b^ | CLQ (K %) | | Obs/Exp^b^ | CLQ (K %) |
| Index rater |  |  |  |  |  | |  |  | |  |  | |  |  | |  |  |
| 1 Disagree completely | 21 | 6.0/4.4 | 1.4 (9.4)^***^ | 9.4/8.8 | 1.1 (2.1)^*^ | | 2.7/4.1 | 0.66 (-8.0)^***^ | | 0.84/1.6 | 0.51 (-6.2)^***^ | | 1.2/1.2 | 1.0 ( 0.5) | | 0.90/0.94 | 0.99 ( -0.0) |
| 2 Disagree | 41 |  |  | 19/18 | 1.1 (3.2)^***^ | | 7.2/8.1 | 0.89 (-3.7)^***^ | | 2.7/3.3 | 0.84 (-2.4)^***^ | | 2.4/2.3 | 1.0 ( 0.1) | | 1.8/1.8 | 0.98 (-0.1) |
| 3 Agree | 19 |  |  |  |  | | 5.2/3.7 | 1.4 ( 10)^***^ | | 2.4/1.5 | 1.6 (7.2)^***^ | | 0.94/1.1 | 0.86 (-1.2) | | 0.75/0.83 | 0.89 (-0.8) |
| 4 Agree completely | 7.8 |  |  |  |  | |  |  | | 1.3/0.61 | 2.1 (9.5)^***^ | | 0.30/0.44 | 0.69 (-1.9) | | 0.30/0.33 | 0.89 (-0.5) |
| 5 Cannot say | 5.6 |  |  |  |  | |  |  | |  |  | | 0.44/0.31 | 1.4 ( 2.7) | | 0.32/0.24 | 1.3 (1.8) |
| 6 Not answered | 4.3 |  |  |  |  | |  |  | |  |  | |  |  | | 0.27/0.18 | 1.4 (2.0) |

* p <0.05; ** p <0.01; *** p <0.001

^a^ Relative frequency of index ratings in each category

^b^ Observed/Expected relative frequency (%) used in the calculation of the colocation quotient (CLQ)

**Table S2. Index – nearest neighbor rater agreement on spaciousness in the close** outdoor environment

|  | Rel | Nearest neighbor rater | | | | | | | | | | | | | |
| --- | --- | --- | --- | --- | --- | --- | --- | --- | --- | --- | --- | --- | --- | --- | --- |
|  | freq^a^ | 1. Disagree completely | | 2. Disagree | | 3. Agree | | 4. Agree completely | | 5. Cannot say | | | 6. Not answered | | |
|  | (%) | Obs/Exp^b^ | CLQ (K %) | Obs/Exp^b^ | CLQ (K %) | Obs/Exp^b^ | CLQ (K %) | Obs/Exp^b^ | CLQ (K %) | | Obs/Exp^b^ | CLQ (K %) | | Obs/Exp^b^ | CLQ (K %) |
| Index rater |  |  |  |  |  |  |  |  |  | |  |  | |  |  |
| 1 Disagree completely | 7.7 | 0.98/0.59 | 1.7 (5.4)^***^ | 2.2/1.7 | 1.3 (2.4)^*^ | 3.0/3.4 | 0.88 (-1.8)^**^ | 0.64/1.3 | 0.50 (-4.3)^***^ | | 0.48/0.39 | 1.2 (1.9) | | 0.39/0.33 | 1.2 ( 1.2) |
| 2 Disagree | 22 |  |  | 5.9/4.8 | 1.2 (5.1)^***^ | 9.5/9.8 | 0.97 (-1.1) | 2.3/3.6 | 0.64 (-7.3)^***^ | | 1.2/1.1 | 1.0 (0.6) | | 0.90/0.95 | 0.94 (-0.3) |
| 3 Agree | 45 |  |  |  |  | 21/20 | 1.1 (3.8)^***^ | 7.1/7.3 | 0.97 (-0.3) | | 2.1/2.2 | 0.95 (-0.4) | | 1.8/1.9 | 0.93 (-0.5) |
| 4 Agree completely | 17 |  |  |  |  |  |  | 4.9/2.7 | 1.8 (17)^***^ | | 0.54/0.83 | 0.65 (-2.7)^*^ | | 0.66/0.71 | 0.93 (-0.4) |
| 5 Cannot say | 5.0 |  |  |  |  |  |  |  |  | | 0.40/0.25 | 1.6 (3.4) | | 0.31/0.22 | 1.4 (2.3) |
| 6 Not answered | 4.3 |  |  |  |  |  |  |  |  | |  |  | | 0.26/0.18 | 1.4 (2.0) |

* p <0.05; ** p <0.01; *** p <0.001

^a^ Relative frequency of index ratings in each category

^b^ Observed/Expected relative frequency (%) used in the calculation of the colocation quotient (CLQ)

**Table S3. Index – nearest neighbor rater agreement on cultural history in the close** outdoor environment

|  | Rel | Nearest neighbor rater | | | | | | | | | | | | | | |
| --- | --- | --- | --- | --- | --- | --- | --- | --- | --- | --- | --- | --- | --- | --- | --- | --- |
|  | freq^a^ | 1. Disagree completely | | 2. Disagree | | 3. Agree | | 4. Agree completely | | | 5. Cannot say | | | 6. Not answered | | |
|  | (%) | Obs/Exp^b^ | CLQ (K %) | Obs/Exp^b^ | CLQ (K %) | Obs/Exp^b^ | CLQ (K %) | | Obs/Exp^b^ | CLQ (K %) | | Obs/Exp^b^ | CLQ (K %) | | Obs/Exp^b^ | CLQ (K %) |
| Index rater |  |  |  |  |  |  |  | |  |  | |  |  | |  |  |
| 1 Disagree completely | 17 | 4.2/3.0 | 1.4 ( 7.4) ^***^ | 6.4/5.8 | 1.1 (2.8)^***^ | 3.4/4.6 | 0.74 (-6.6)^***^ | | 0.81/1.6 | 0.50 (-5.6)^***^ | | 1.7/1.6 | 1.1 ( 1.0) | | 0.81/0.75 | 1.1 ( 0.5) |
| 2 Disagree | 33 |  |  | 12/11 | 1.1 (4.0)^***^ | 8.2/8.8 | 0.93 (-2.6)^***^ | | 2.1/3.1 | 0.68 (-4.6)^***^ | | 2.9/3.1 | 0.97 ( -0.5) | | 1.4/1.4 | 0.98 (-0.3) |
| 3 Agree | 27 |  |  |  |  | 8.2/7.1 | 1.2 (6.4)^***^ | | 3.2/2.5 | 1.3 ( 5.7)^***^ | | 2.2/2.5 | 0.92 (-1.3) | | 1.0/1.1 | 0.90 (-0.9) |
| 4 Agree completely | 9.4 |  |  |  |  |  |  | | 1.9/0.88 | 2.2 (14)^***^ | | 0.69/0.88 | 0.80 (-2.0) | | 0.35/0.0 | 0.87 (-1.1) |
| 5 Cannot say | 9.1 |  |  |  |  |  |  | |  |  | | 1.1/0.88 | 1.3 ( 3.3)^*^ | | 0.48/0.40 | 1.2 ( 1.1) |
| 6 Not answered | 4.3 |  |  |  |  |  |  | |  |  | |  |  | | 0.25/0.18 | 1.4 ( 1.1) |

* p <0.05; ** p<0.01; *** p <0.001

^a^ Relative frequency of index ratings in each category

^b^ Observed/Expected relative frequency (%) used in the calculation of the colocation quotient (CLQ)
